# Characterization of Synthetic Chikungunya Viruses Based on the Consensus Sequence of Recent E1-226V Isolates

**DOI:** 10.1371/journal.pone.0071047

**Published:** 2013-08-01

**Authors:** Florine E. M. Scholte, Ali Tas, Byron E. E. Martina, Paolo Cordioli, Krishna Narayanan, Shinji Makino, Eric J. Snijder, Martijn J. van Hemert

**Affiliations:** 1 Molecular Virology Laboratory, Department of Medical Microbiology, Leiden University Medical Center, Leiden, The Netherlands; 2 Department of Viroscience, Erasmus Medical Center, Rotterdam, The Netherlands; 3 Istituto Zooprofilattico Sperimentale della Lombardia e dell’Emilia Romagna, Brescia, Italy; 4 Department of Microbiology and Immunology, University of Texas Medical Branch, Galveston, Texas, United States of America; Agency for Science, Technology and Research - Singapore Immunology Network, Singapore

## Abstract

Chikungunya virus (CHIKV) is a mosquito-borne alphavirus that re-emerged in 2004 and has caused massive outbreaks in recent years. The lack of a licensed vaccine or treatment options emphasize the need to obtain more insight into the viral life cycle and CHIKV-host interactions. Infectious cDNA clones are important tools for such studies, and for mechanism of action studies on antiviral compounds. Existing CHIKV cDNA clones are based on a single genome from an individual clinical isolate, which is expected to have evolved specific characteristics in response to the host environment, and possibly also during subsequent cell culture passaging. To obtain a virus expected to have the general characteristics of the recent E1-226V CHIKV isolates, we have constructed a new CHIKV full-length cDNA clone, CHIKV LS3, based on the consensus sequence of their aligned genomes. Here we report the characterization of this synthetic virus and a green fluorescent protein-expressing variant (CHIKV LS3-GFP). Their characteristics were compared to those of natural strain ITA07-RA1, which was isolated during the 2007 outbreak in Italy. In cell culture the synthetic viruses displayed phenotypes comparable to the natural isolate, and in a mouse model they caused lethal infections that were indistinguishable from infections with a natural strain. Compared to ITA07-RA1 and clinical isolate NL10/152, the synthetic viruses displayed similar sensitivities to several antiviral compounds. 3-deaza-adenosine was identified as a new inhibitor of CHIKV replication. Cyclosporin A had no effect on CHIKV replication, suggesting that cyclophilins -opposite to what was found for other +RNA viruses- do not play an essential role in CHIKV replication. The characterization of the consensus sequence-based synthetic viruses and their comparison to natural isolates demonstrated that CHIKV LS3 and LS3-GFP are suitable and representative tools to study CHIKV-host interactions, screen for antiviral compounds and unravel their mode of action.

## Introduction

Chikungunya virus (CHIKV) re-emerged in 2004 and has caused unprecedented outbreaks in Asia and Africa since 2005. The estimated number of cases exceeds 2 million and over a thousand infected travelers have returned to Europe and the USA since 2006 [1], [2]. CHIKV generally causes a fever that resolves within several days, a maculopapular rash, and a characteristic arthralgia that can be extremely painful and may persist for months. During the recent outbreaks also more severe clinical manifestations have been reported occasionally, such as neurological complications and even deaths, usually in the elderly, patients with underlying conditions, and newborns [3], [4]. A licensed vaccine or specific antiviral therapy are currently not available.

CHIKV is an alphavirus with an 11.7 kb positive-stranded RNA genome that contains two open reading frames (ORFs). The 5′ ORF encodes the nonstructural polyproteins P123 and P1234. The latter results from translational read-through of an opal termination codon that is present at the end of the nonstructural protein (nsP) 3 coding sequence of most CHIKV isolates. Assuming that CHIKV follows the typical alphavirus life cycle, proteolytic processing of the nonstructural polyproteins by the protease domain in nsP2 will ultimately lead to the release of nsP1, nsP2, nsP3, and nsP4. These nsPs and their precursors possess a variety of functions and the enzymatic activities, including protease, helicase, methyltransferase, and RNA-dependent RNA polymerase (RdRp) activity that drive CHIKV replication [5]. In addition to replication of its genomic RNA, CHIKV also transcribes a subgenomic (sg) RNA encoding a precursor polyprotein that is processed by viral and cellular proteases into the structural proteins C, E3, E2, 6K and E1. CHIKV nsPs will - presumably together with host factors - assemble into replication and transcription complexes (RTCs) that associate with membrane structures derived from the plasma membrane and/or endosomes, as observed for other alphaviruses [5]–[7].

The CHIKV strains that emerged during the 2005–2006 outbreaks had acquired a mutation (A226V) in the E1 envelope glycoprotein, which facilitated transmission of the virus via a new vector, the Asian tiger mosquito *Aedes albopictus*, and consequently dramatically increased the epidemic potential of CHIKV [8], [9]. Later studies suggested that recent Indian and Indian Ocean epidemics have emerged separately as the result of at least three independent events, and that convergent evolution of East-Central-South African lineage strains in different geographical regions ultimately led to the emergence of strains with the A226V substitution in E1 [10]–[13]. More recently, other amino acid positions and epistatic interactions were also shown to play an important role in the emergence of these new CHIKV variants, which are now even replacing endemic strains that have been circulating in Asia for decades [14]. *Aedes albopictus* also thrives in more temperate climates and its geographical distribution has rapidly expanded. Over the past decades, parts of southern Europe and large areas of the USA have been invaded by this mosquito, providing imported cases of CHIKV with a competent mosquito vector, thus paving the road for outbreaks in non-endemic-areas such as the USA and Europe. Indeed, autochthonous infections have been reported from Italy in 2007 and France in 2010 [15], [16]. The recent and ongoing CHIKV outbreaks are characterized by their rapid geographical spread, high numbers of infected people and high morbidity, emphasizing the need to gain more insight into the replicative cycle of this important human pathogen.

Infectious cDNA clones of viruses have become invaluable tools that allow reverse genetics studies to elucidate the contribution of specific amino acids or RNA structures to viraemia, virulence, antigenicity, replication kinetics, interactions with host factors, adaptation to new vectors, and many other aspects of the viral life cycle. The use of cDNA clones is also instrumental in mechanism of action studies to pinpoint the viral target of antiviral compounds by selecting for and genotyping compound-resistant viruses, followed by reverse engineering of the identified mutations to assess their individual phenotypic contribution to resistance. Finally, the generation of cDNA clones of reporter viruses, like those expressing green fluorescent protein (GFP), greatly facilitates high throughput screening, e.g. for antiviral compounds or host factors that affect replication.

Several CHIKV cDNA clones have been constructed in the past, which - except for the West African lineage strain 37997 strain that was isolated from a mosquito - were all based on clinical isolates from infected humans [17]–[23]. Each natural isolate is expected to have evolved its own specific characteristics in terms of sequence, virulence and virus-host interactions as a result of specific selective pressures within the infected host (tissue) and possibly also during subsequent passaging in cell culture. Intrahost evolution and quasispecies diversity is expected to be substantial, especially compared to the relatively low level of interhost variation when the consensus sequences of CHIKV genomes isolated from different hosts are aligned. The low level of interhost variation is a typical trait of arboviruses, due to evolutionary constraints imposed by the alternating replication in vertebrate and arthropod hosts. A recent study on the distantly related Ross River virus indeed reported a high level of intrahost diversity [24]. The existing CHIKV molecular clones can be considered to represent a single individual genome (or fragments of several individual genomes) out of the whole spectrum of viruses present in the CHIKV quasispecies population that has been shaped by intrahost evolution and probably a complex set of environmental factors. In contrast, most deposited CHIKV genome sequences represent the consensus (or master sequence) of a viral quasispecies population.

To obtain a virus that - in terms of virulence, sensitivity to antiviral compounds, and CHIKV-host interactions - is expected to have the general characteristics of the E1-226V CHIKV strains that were circulating during the 2005–2009 outbreaks, we have constructed a completely synthetic CHIKV cDNA clone based on the consensus sequence of the aligned genomes of these recent isolates. This new infectious clone, CHIKV LS3 (Leiden Synthetic 3), and a variant that expresses the eGFP reporter gene under control of a duplicated subgenomic promoter (CHIKV LS3-GFP), were created by custom DNA synthesis.

The properties and replicative cycle of the new synthetic viruses were characterized in detail, and comparison with a field isolate (ITA07-RA1) from the 2007 CHIKV outbreak in Italy demonstrated that they have similar characteristics. The sensitivity of LS3 to a number of antiviral compounds was compared to those of ITA07-RA1 and clinical isolate NL10/152. All compounds tested had a similar antiviral activity against LS3 and the natural isolates. These experiments also identified 3-deaza-adenosine as a novel inhibitor of CHIKV replication. This study describes a detailed characterization of the CHIKV replication cycle at the molecular level and demonstrates that a new synthetic infectious clone-derived virus is a useful and representative tool to gain more insight into the replicative cycle of CHIKV, its interactions with the host, and the mode of action of antiviral compounds, which should aid in the development of antiviral strategies against this important human pathogen.

## Materials and Methods

### Cells and Viruses

Vero E6, *Ae. albopictus* C6/36 [25] and 293/ACE2 cells [26] were maintained in Dulbecco’s modified Eagle’s medium (DMEM; Lonza), supplemented with 8% fetal calf serum (FCS; PAA), 2 mM L-glutamine, 100 IU/ml of penicillin and 100 µg/ml of streptomycin. 293/ACE2 cells were grown in the presence of 12 µg/ml blasticidin (PAA) and C6/36 medium was supplemented with non-essential amino acids (Lonza). BHK-21 cells were cultured in Glasgow’s Modified Eagles Medium (Gibco) supplemented with 7.5% FCS, 10 mM HEPES pH 7.4, 8% tryptose phosphate broth (Gibco), and antibiotics. The mammalian cell lines were grown at 37°C and C6/36 cells at 30°C in 5% CO_2_. CHIKV strain ITA07-RA1 (GenBank accession number EU244823) was isolated from *Ae. albopictus* during the 2007 outbreak in Ravenna, Italy, and was passaged twice on BHK-21 cells. CHIKV NL10/152 (GenBank KC862329) was isolated at the Erasmus Medical Center in Rotterdam from the serum of an infected traveler that returned from Indonesia and was passaged twice on Vero cells. Working stocks of CHIKV were routinely produced in Vero E6 cells at 37°C, typically yielding titers of ∼10^7^ PFU/ml. Infections were performed in Eagle’s minimal essential medium (EMEM; Lonza) with 25 mM HEPES (Lonza) supplemented with 2% FCS, L-glutamine, and antibiotics. After 1 h, the inoculum was replaced with fresh culture medium. All procedures with live CHIKV were performed in a biosafety level 3 facility at the Leiden University Medical Center.

### Construction of Synthetic CHIKV Full-length cDNA Clones

**Figure 1:**
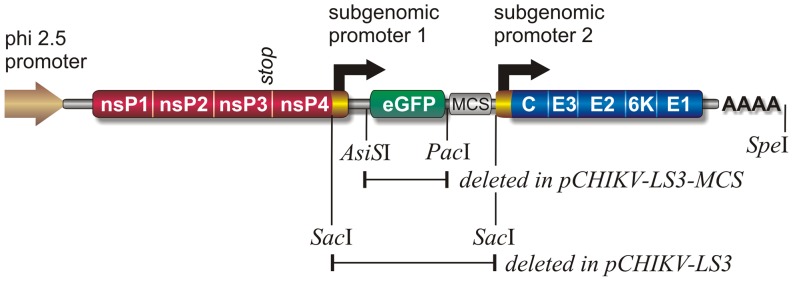
Schematic overview of the synthetic infectious clone pCHIKV-LS3-GFP. The ‘wild type’ full-length clone pCHIKV-LS3, which lacks the eGFP reporter gene, was generated by deleting the *Sac*I fragment; the variant pCHIKV-LS3-MCS, containing a multiple cloning site (MCS) preceded by subgenomic promoter 1, was generated by removing the *Asi*SI-*Pac*I fragment.

A cDNA clone of the synthetic CHIKV strain LS3-GFP, which contains a duplicated subgenomic promoter and expresses the eGFP reporter gene, was designed *in silico* as described in the results section. Three DNA fragments together forming a cDNA copy of CHIKV LS3-GFP were chemically synthesized (GeneArt, Germany). Using standard cloning techniques, these fragments were assembled and cloned into the *Asc*I-*Not*I sites of vector pUC19AN, a pUC19-derived plasmid in which the original polylinker was replaced by one with *Asc*I-*Nco*I-*Eco*RV-*Xho*I-*Not*I sites. The resulting plasmid (pCHIKV-LS3-GFP) contains the genomic cDNA of CHIKV LS3-GFP directly downstream of a phi2.5 promoter and followed by a unique *Spe*I linearization site for DNA linearization prior to *in vitro* transcription. The ‘wild type’ synthetic pCHIKV-LS3 construct was made by deleting the 920 bp eGFP-containing *Sac*I fragment from pCHIKV-LS3-GFP (Fig. 1). A third variant with a duplicated subgenomic promoter and a multiple cloning site behind subgenomic promoter 1 (pCHIKV-LS3-MCS), which allows the introduction of e.g. a reporter gene, was generated by removing the 737 bp *Asi*SI-*Pac*I fragment from pCHIKV-LS3-GFP. The constructs were verified by sequencing.

### *In vitro* Transcription and RNA Transfection

RNA was transcribed from plasmids with the phi2.5 promoter [27] using the AmpliScribe T7 high yield transcription kit (Epicenter), the m^7^GpppA RNA cap structure analog (NEB) and 0.7 µg of template DNA that had been linearized with *Spe*I. After a 3-h reaction at 37°C, template DNA was digested with DnaseI and RNA was purified by precipitation with 7.5 M LiCl (Ambion). The concentration of *in vitro* transcribed RNA was determined with a NanoDrop spectrophotometer (Thermo Scientific) and its integrity was checked by agarose gel electrophoresis. BHK-21 cells (2×10^6^) were electroporated with 1 µg of RNA using program T-20 of the Amaxa Nucleofector and Kit T (Lonza) according to the manufacturer's instructions. Electroporated cells were plated in 6-well clusters and incubated at 37°C in the same medium used for CHIKV infection experiments.

### Sequencing of CHIKV Genomes

CHIKV RNA was isolated from virions using the QIAamp Viral RNA mini kit. Four overlapping amplicons were generated by a two-step reverse transcriptase (RT) PCR. In the first step cDNA was synthesized using RevertAid H Minus Reverse transcriptase (Fermentas) and primers AT-39 (GACTGCAGATGCCCGCCATT), AT-41 (CGCTCGGTCCAGGCAACTCT), AT-43 (CGTGGTGTTTGCCAACAGGC), or AT-52 (CGCCGTTTTTTTTTTTTTTTTTTTTTTTTT). In the second step 4 PCR products were generated using combinations of primers AT-38 (ATGGCTGCGTGAGACACACG) and AT-39, AT-40 (TGCACCCAAGTGTACCACAA) and AT-41, AT-42 (CAGGAGAGTGCATCCATGGC) and AT-43, or AT-44 (GAATGCGCGCAGATACCCGT) and AT-52. The resulting RT-PCR products were purified and directly sequenced (50 ng template) using the BigDye Terminator Cycle Sequencing Kit v1.1 (Applied Biosystems) and a 3130 Genetic Analyzer automatic sequencer (Applied Biosystems). PCR conditions and primer sequences are available upon request.

### Virus Titration and Infectious Center Assay

Viral titers were determined by plaque assay on Vero E6 cells. Six-well clusters containing confluent monolayers of Vero E6 cells were incubated with 0.5-ml volumes of 10-fold serial dilutions of CHIKV-containing samples. After a 1-h incubation at 37°C, the inoculum was replaced with 2 ml of DMEM containing 1.2% Avicel RC-581 (FMC BioPolymer), 2% FCS, 25 mM HEPES, and antibiotics. After a 66-h incubation at 36°C, monolayers were fixed with 3.7% formaldehyde in PBS and plaques were visualized by crystal violet staining. For infectious center assays 10-fold serial dilutions of electroporated cells were added to 6-well clusters already containing a monolayer of 1×10^6^ BHK-21 cells per well. After a 1-h incubation at 37°C, a DMEM/Avicel overlay was applied and cells were incubated at 37°C for 48 h. Plaques were visualized as described above.

### RNA Isolation, Denaturing Agarose Electrophoresis and in-gel Hybridization

Total RNA was isolated from 7×10^5^ cells by lysis in 0.5 ml of 20 mM Tris-HCl (pH 7.4), 100 mM LiCl, 2 mM EDTA, 5 mM DTT, 5% (w/v) lithium dodecyl sulfate, and 100 µg/ml proteinase K. After acid phenol (Ambion) extraction, RNA was precipitated with isopropanol, washed with 75% ethanol, and dissolved in 1 mM sodium citrate (pH 6.4). Samples containing RNA from 4.7×10^4^ cells were mixed with 3 volumes of 67% formamide, 23% formaldehyde, 6.7% glycerol, 13 mM MOPS (pH 7.2), 6.7 mM NaAc, 2.7 mM EDTA, 0.07% SDS, and 0.03% bromophenol blue. After denaturation for 15 min at 75°C, RNA was separated in 1.5% denaturing formaldehyde-agarose gels using the MOPS buffer system as described [28]. RNA molecules were detected by direct hybridization of the dried gel with ^32^P-labeled oligonucleotides essentially as described previously [29]. Positive-stranded genomic and subgenomic CHIKV RNAs were visualized with probe CHIKV-hyb4 (5′-TGTGGGTTCGGAGAATCGTGGAAGAGTT-3′) that is complementary to the 3′ end of the genome. Negative-stranded RNA was detected with probe CHIKV-hyb2 (5′-AACCCATCATGGATCCTGTGTACGTGGA-3′) that is complementary to the 3′ end of the minus strand. 18S ribosomal RNA (loading control) was detected with the oligonucleotide probe 5′-ATGCCCCCGGCCGTCCCTCT-3′. Probes (10 pmol) were labeled with 10 µCi [γ-^32^P]ATP (PerkinElmer) in a 1h reaction using 10 U of T4 polynucleotide kinase (Invitrogen) in 10 µl of the supplied forward reaction buffer (Invitrogen). Prehybridization (1 h) and hybridization (overnight) were done at 55°C in 5× SSPE (0.9 M NaCl, 50 mM NaH_2_PO_4_, 5 mM EDTA, pH 7.4), 5× Denhardt’s solution, 0.05% SDS, and 0.1 mg/ml homomix I. Hybridized gels were washed twice in 5× SSPE with 0.05% SDS before they were exposed to Storage Phosphor screens. After scanning with a Typhoon-9410 scanner (GE Healthcare), quantification of RNA levels was done with Quantity One v4.5.1 (Biorad) and corrections for loading variations were made based on the quantity of 18S ribosomal RNA in the same lane. The results of two or three independent experiments were quantified (one representative experiment is shown in figures).

### Western Blot Analysis

Total protein samples were prepared by lysing 7×10^5^ cells in 0.5 ml of 4× Laemmli sample buffer (100 mM Tris-HCl, pH 6.8, 40% glycerol, 8% SDS, 40 mM DTT, 0,04 mg/ml bromophenol blue). Proteins were separated by SDS-PAGE in 12% polyacrylamide gels and were transferred to Hybond-LFP membranes (GE Healthcare) by semi-dry blotting. After blocking with 1% casein (Sigma) in PBS with 0.1% Tween-20 (PBST), membranes were incubated overnight with rabbit antisera against CHIKV nsP1 (raised using the peptide EVEPRQVTPNDHAN), nsP4 (raised using the peptide ASSRSNFEKLRGPV) or E2 [30] in PBST with 0.5% casein. Mouse monoclonal antibodies against β-actin (Sigma), or the transferrin receptor (Zymed) were used for detection of loading controls. Biotin-conjugated swine-α-rabbit (DAKO) or goat-α-mouse (DAKO), and Cy3-conjugated mouse-α-biotin (Jackson) were used for fluorescent detection of the primary antibodies with a Typhoon-9410 scanner (GE Healthcare).

### Metabolic Labeling with ^3^H-uridine

At various time points post infection approximately 2×10^5^ CHIKV-infected or mock-infected 293/ACE2 cells in 12-well clusters were incubated with 40 µCi of ^3^H-uridine in medium and incorporation was allowed to proceed for 60 minutes at 37°C. Total RNA was isolated and analyzed in a denaturing agarose gel as described above. For fluorographic detection of ^3^H-labeled RNA, the gel was soaked in methanol for 1 hour (one change) and then incubated with 3% 2,5-diphenyloxazole in methanol for at least 3 hours. After incubation in milliQ for 30 minutes, the gel was dried and a Fuji RX film was placed on top. Films were developed after a 1–4 day exposure at −80°C and scanned with a Biorad GS-800 densitometer. To check equal sample loading, the gel was hybridized with a ^32^P-labeled 18S ribosomal RNA-specific probe as described above. In addition, incorporation of ^3^H-uridine into RNA was quantified by analyzing 2-µl samples of isolated total RNA with a liquid scintillation counter (Beckman LS 6500 IC). In control samples, cellular transcription was inhibited by adding Actinomycin D (Sigma) to a final concentration of 5 µg/ml.

### Metabolic Labeling of Proteins with ^35^S-methionine and ^35^S-cysteine

At various time points post infection approximately 2×10^5^ CHIKV-infected or mock-infected 293/ACE2 cells in 12-well clusters were starved in DMEM lacking L-methionine and L-cysteine (Invitrogen) for 30 min., and subsequently incubated with 44 µCi EasyTag EXPRESS ^35^S protein labeling mix (PerkinElmer) for 30 min. Total protein samples were analyzed by SDS-PAGE as described above. Gels were stained with Coomassie to check equal sample loading and ^35^S-labeled proteins were detected by drying the gels and exposing them to a Storage Phosphor screen, which was scanned 1–2 days later with a Typhoon-9410 scanner (GE Healthcare).

### Indirect Immunofluorescence Microscopy

CHIKV- or mock-infected Vero E6 cells grown on coverslips were fixed with 3% paraformaldehyde in PBS. After quenching with 10 mM glycine in PBS, cells were permeabilized with 0.1% Triton in PBS for 10 min. and coverslips were incubated with primary antibodies diluted in PBS with 5% FCS for 1 h. Double-stranded RNA was detected with a 1∶200 dilution of mouse monoclonal antibody J2 (English & Scientific Consulting). CHIKV E2 was visualized with a 1∶8000 dilution of a polyclonal rabbit antiserum [30]. Detection of primary antibodies was done with donkey-α-mouse-Cy3, goat-α-rabbit-Cy3 or goat-α-rabbit-Alexa488 (1∶500; Jackson). Nuclei were stained with Hoechst 33342. The coverslips were mounted with Prolong (Invitrogen) and analyzed using an Axioskop2 Mot Plus fluorescence microscope with Axiocam HRc camera and AxioVision software (Zeiss).

### Virus Neutralization Assay

Mouse monoclonal antibodies raised against CHIKV particles of strain ITA07-RA1 (IZSLER, Brescia, Italy) were heat-inactivated for 30 min. at 56°C. Two-fold serial dilutions of the neutralizing monoclonal antibody 1H7 and non-neutralizing control antibody 3H9 [31] were incubated with an equal volume of medium containing 100 PFU of CHIKV. These mixtures were incubated for 60 min. at 37°C and transferred to 96-well clusters containing 2×10^4^ Vero E6 cells per well. After incubation at 37°C for 2 days, the wells were fixed with 3.7% formaldehyde and CPE was detected by staining with crystal violet.

### Antiviral Compound Assays

Chloroquine, 6-aza-uridine and ribavirin were dissolved in PBS. Cyclosporin A and 3-deaza-adenosine were dissolved in DMSO. Mycophenolic acid was dissolved in ethanol. All compounds were obtained from Sigma. For CPE reduction assays, 96-well clusters with ∼1×10^4^ Vero E6 cells/well were incubated with 50 PFU of virus per well, corresponding to a multiplicity of infection (MOI) of 0.005, and 2-fold serial dilutions of the compound in medium. Wells without cells, uninfected cells, infected untreated cells and infected cells treated with solvent alone were included as controls. Four days post-infection cell viability was assessed using the CellTiter 96® AQueous Non-Radioactive Cell Proliferation Assay (Promega). CPE reduction experiments with ribavirin were done with BHK-21 cells in a similar way, except that viability was assessed 2 days post infection. For eGFP reporter gene assays, ∼1×10^4^ Vero E6 cells/well in black 96-well plates were infected with CHIKV LS3-GFP at an MOI of 0.05. After a 42-h incubation in medium containing the compound, the cells were fixed with 3% paraformaldehyde in PBS. eGFP expression was quantified using a Berthold Mithras LB 940 plate reader, with excitation and emission wavelengths of 485 and 535 nm, respectively. The fluorescence in wells containing mock-infected cells was used to correct for background signal. IC_50_ and CC_50_ values were calculated with GraphPad Prism 5 using the nonlinear regression model.

### Mouse Experiments

All animal experiments described in this paper were carried out in the BSL3 facilities of the Erasmus Medical Center in accordance with the Dutch guidelines for animal experimentation and were approved by the institute’s independent animal ethics committee. Twelve-day old C57BL/6 mice were injected intraperitoneally with 100 TCID_50_ of CHIKV S27, CHIKV LS3 or CHIKV LS3-GFP. After the challenge the mice were monitored daily for signs of illness or death. The infection was considered lethal when the animals reached humane end-points and needed to be euthanized. Viral RNA was extracted from brain samples using the automated MagnaPure method (Total nucleic acid isolation kit, Roche Diagnostics, the Netherlands) according to the manufacturer’s instructions, and quantified using a one-step RT-PCR TaqMan protocol (EZ-kit, Applied Biosystems) and an ABI PRISM 7500 detection instrument. The primers and probe used for CHIKV RNA quantification were essentially as described [32] except that probe Fam-CCAATGTCTTCAGCCTGGACACCTTT-Tamra was used. Dilutions of virus suspensions of known titer were included to make a calibration curve, which was used to express results as TCID_50_ equivalents per gram of brain tissue.

### Ethics Statement

All animal experiments described in this paper were carried out in the BSL3 facilities of the Erasmus Medical Center in accordance with the Dutch guidelines for animal experimentation. A Dutch Government-approved and independent animal experimentation ethical review committee (Stichting DEC Consult) approved the animal studies (permit nr. EMC2838/122-12-29).

### Nucleotide Sequence Accession Numbers

The GenBank accession numbers for the full-length cDNA clones pCHIKV-LS3, pCHIKV-LS3-GFP and pCHIKV-LS3-MCS are JX911334, JX911335, and JX911336 respectively. The Genbank accession numbers for the genomic RNA sequences of CHIKV LS3, LS3-GFP, LCS3-MCS and NL10/152 are KC149888, KC149887, KC149889, and KC862329, respectively.

## Results

### *In silico* Design and Construction of Synthetic CHIKV Full-length cDNA Clones

The complete genomes of the 13 CHIKV strains carrying the E1-A226V mutation (Table 1) that were available in GenBank at the time of *in silico* design (November 2009) were aligned using MAFFT [33] and the resulting consensus sequence formed the basis for the synthetic full-length cDNA clones. A 40 nucleotide polyA tail was added to the 3′ end of the consensus sequence and an A7435G point mutation was introduced to create a translationally silent *SacI* restriction site required for cloning. The virus encoded by the resulting sequence was termed CHIKV LS3 (GenBank accession KC149888). Variants containing a duplicated subgenomic promoter and a multiple cloning site (CHIKV LS3-MCS; GenBank KC149889) or an eGFP reporter gene (CHIKV LS3-GFP; GenBank KC149887) were also designed. The eGFP reporter gene was placed under control of the native subgenomic promoter and upstream of a second subgenomic promoter that drives expression of the viral structural polyprotein, as this configuration was previously reported to result in a more stable reporter gene expression [18]. The CHIKV cDNAs were placed downstream of a phi2.5 T7 promoter, and a unique *SpeI* site for linearization prior to *in vitro* transcription directly followed the polyA tail. The phi2.5 promoter was used because the 5′ ends of capped transcripts generated from this promoter with T7 polymerase and the m^7^Gppp**A** cap analog are identical to the 5′ end of the genomes of naturally occurring CHIKV strains. In contrast, capped RNAs generated by *in vitro* transcription from the frequently used SP6 promoter will contain m^7^Gppp**G** at their 5′ terminus, i.e. will contain an additional 5′-terminal G residue. However, existing CHIKV cDNA clones that contain the SP6 promoter also efficiently yield infectious virus and it is assumed that the additional 5′-terminal G residue is removed during subsequent rounds of replication. In line with this, *in vitro* transcribed RNA from pCHIKV-LS2, a variant of plasmid pCHIKV-LS3 in which the phi2.5 promoter was replaced with the SP6 promoter also yielded infectious virus.

**Table 1:** CHIKV E1-226V strains that were aligned to produce the CHIKV LS3 consensus sequence.

CHIKV strain	Origin	Year	GenBank accession
BNI-CHIKV_899	Mauritius	2006	FJ959103.1
D570/06	Mauritius	2006	EF012359.1
LR2006_OPY1	La Reunion	2006	DQ443544.2
TM25	Mauritius	2006	EU564334.1
Wuerzburg 1	Mauritius	2006	EU037962.1
DRDE-07	India	2007	EU372006.1
ITA07-RA1	Italy	2007	EU244823.2
RGCB80/KL07	India	2007	GQ428212.1
RGCB120/KL07	India	2007	GQ428213.1
0810aTw	Bangladesh	2008	FJ807898.1
0810bTw	Malaysia	2008	FJ807899.1
RGCB355/KL08	India	2008	GQ428214.1
RGCB356/KL08	India	2008	GQ428215.1

Plasmid pCHIKV-LS3-GFP, the infectious clone encoding the eGFP-expressing reporter virus, was created by assembling the chemically synthesized DNA fragments as described in the Materials and Methods section. Plasmid pCHIKV-LS3, the infectious clone encoding the synthetic ‘wild type’ strain CHIKV LS3, and plasmid pCHIKV-LS3-MCS were generated from pCHIKV-LS3-GFP by deleting specific restriction fragments, as described in Materials and Methods (Fig. 1).

In the original alignment, strains DRDE-07 (GenBank U372006) and D570/06 (GenBank EF012359) shared the highest sequence similarity with LS3, with 3 amino acid differences (Table 2). However, a BLAST search performed in March 2013, three years after the design of CHIKV LS3, and alignment of the retrieved complete CHIKV genomes revealed that strains IND-06-AP3 (GenBank EF027134), IND-GJ53 (GenBank FJ000065), and CHIK31 (GenBank EU564335) share the highest degree of nucleotide sequence identity with CHIKV LS3 (>99.9%), with only 5–7 nucleotide differences respectively (table S1). Interestingly, these Indian strains were not included in the original alignment on which the LS3 sequence was based, as they do not contain the E1-A226V mutation (Table 1). However, nsP1234 of LS3 is identical to that of IND-06-AP3. At the amino acid level, CHIKV LS3 differs at 4 positions from LR2006_OPY1 and at 3 positions from ITA07-RA1 (Table 2).

**Table 2:** Comparison of the amino acid sequences of CHIKV LS3 and various natural isolates.

	nspP1								nsP2	nsP3			nsP4	C		E2		E1	
position in protein	128	157	184	230	314	326	376	391	539	155	376	472	88	23	27	191	252	4	226
LS3	K	H	M	G	M	V	M	F	L	A	T	S	R	S	V	T	K	V	V
IND-06-AP3	.	.	.	.	.	.	.	.	.	.	.	.	.	.	.	.	.	.	A
IND-MH51	.	.	.	.	.	.	.	L	.	.	.	.	.	.	.	.	.	.	A
CHIK31	.	.	.	.	.	.	.	.	.	.	.	N	.	.	I	.	.	.	A
DRDE-07	.	.	T	.	L	.	.	.	.	.	.	.	.	.	.	.	.	A	.
D570/06	T	.	.	.	.	.	T	.	.	.	.	.	.	P	.	.	.	.	.
ITA07-RA1	.	.	.	R	.	.	.	.	.	.	I	.	.	.	I	.	.	.	.
LR2006_OPY1	T	.	.	.	.	M	T	.	.	.	.	.	.	P	.	.	.	.	.
NL10/152	.	Y	.	.	.	.	.	.	S	T	.	.	S	.	I	S	Q	.	.

The CHIKV LS3 amino acid sequences were aligned with those of several highly similar natural isolates, and clinical isolate NL10/152. Only amino acid differences are indicated and identity is represented by dots. The numbering is based on the sequence of LS3, which is also equal to that of LR2006_OPY1. It is important to note that - like all other CHIKV strains in this table - clinical isolate LR2006-OPY1 (Genbank DQ443544) also contains an opal stop codon in the 3′-end of the nsP3 coding region, while its infectious clone with Genbank accession number EU224268 lacks this stop codon.

### Growth Kinetics of Synthetic CHIKV Strains and Comparison to a Natural Isolate

To determine whether infectious virus could be generated from the synthetic CHIKV clones, *in vitro* transcribed RNA was electroporated into BHK-21 cells. Strong eGFP fluorescence was readily detected 16 h after transfection of CHIKV LS3-GFP RNA. For CHIKV LS3 and LS3-GFP RNA specific infectivities of approximately 10^5^ PFU/µg of RNA were found in infectious center assays, which is similar to what has been found for other CHIKV cDNA clones [17], [18]. Virus titers in cell culture supernatants 16 h after electroporation, were generally in the range of 10^5^–10^6^ PFU/ml. This is lower than the peak viral titers that are obtained during infection experiments, but can be explained by the early time point of harvesting and the fact that not all cells were transfected. As expected, electroporation of BHK-21 cells with uncapped CHIKV RNA did not result in the release of infectious virus.

To assess the stability of eGFP reporter expression, CHIKV LS3-GFP was serially passaged (MOI 0.5) in both 293/ACE2 and Vero E6 cells. Virus harvested during each passage was used to infect Vero E6 cells at an MOI of 0.2 and immunofluorescence microscopy revealed that at passage 10, over 95% of the E2-positive foci still displayed robust eGFP expression. Sequencing of cDNA obtained by RT-PCR amplification of RNA extracted from extracellular virions revealed that, after 3 passages on Vero E6 cells, the consensus genome sequence of CHIKV LS3-GFP was identical to the original *in silico* designed sequence. These results demonstrated that the synthetic viruses are viable, genetically stable, and able to retain stable expression of the reporter gene.

Since we aim to use CHIKV in siRNA screens and proteomics studies to identify host factors involved in replication, various human cell lines were evaluated for their ability to support CHIKV replication. CHIKV LS3-GFP was able to productively infect HeLa, MRC-5, Huh7, 293, and 293/ACE2 cells (data not shown). Infection of HeLa and Huh7 cells was not very efficient and these cells were therefore not used for any further experiments. 293/ACE2 cells were selected for this study, as they supported high levels of CHIKV LS3-GFP replication, could be efficiently transfected with siRNAs, and - unlike regular 293 cells - adhered well to tissue culture plastics. 293/ACE2 cells stably express angiotensin-converting enzyme 2 (ACE2), the receptor for SARS-coronavirus. ACE2 expression is not required for CHIKV infection, but these cells were chosen because of the aforementioned advantages and the fact that they have been previously used in our lab in siRNA screens for host factors that affect corona- and arterivirus replication ([34]; de Wilde et al. submitted; Wannee et al., in preparation). Using these cells in similar siRNA screens with CHIKV and other alphaviruses would allow direct comparison of data sets with those obtained for corona- and arteriviruses, which could lead to the identification of common (broad spectrum) pro- and antiviral host factors.

**Figure 2:**
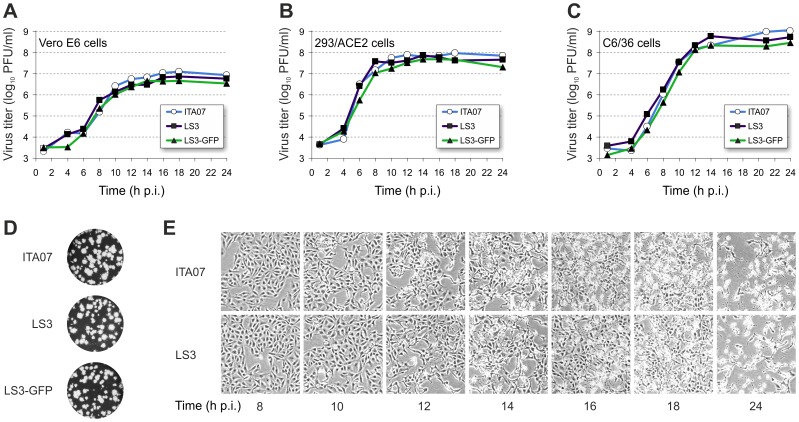
Growth kinetics of CHIKV LS3, LS3-GFP and ITA07-RA1 on various cell lines. Growth kinetics of CHIKV on Vero E6 (A), 293/ACE2 (B) and mosquito C6/36 cells (C). Cells were infected at an MOI of 5 and the viral progeny titers in the supernatant were determined at various time points post infection. (D) Plaque morphology of ITA07, LS3 and LS3-GFP on Vero E6 cells. (E) Induction of CPE by ITA07 (upper panel) and LS3 (lower panel) on Vero E6 cells at different time points post infection.

To determine whether the synthetic viruses behave like natural isolates, their growth kinetics in Vero E6, 293/ACE2, and C6/36 cells were compared to those of ITA07-RA1, which was isolated during the 2007 CHIKV outbreak in Italy (Fig. 2A–C). The growth curves of CHIKV LS3 on all three cell lines were found not to differ significantly from those of ITA07-RA1, with virus titers reaching a maximum 14–18 h post infection (p.i.). Peak virus titers on mosquito cells were approximately 1-log higher than those on mammalian cells. CHIKV LS3-GFP replicated slightly slower than the other viruses in all three tested cell lines, which is not unusual for recombinant reporter viruses. eGFP expression could be detected as early as 6 h p.i. and peaked around 22 h p.i. The plaque morphology of the synthetic viruses was similar to that of ITA07-RA1 (Fig. 2D). CHIKV LS3 induced a cytopathic effect (CPE) indistinguishable from the natural isolate. On Vero E6 cells early signs of CPE started to appear around 12 h p.i. and CPE was complete by 24 h p.i. (Fig. 2E).

**Figure 3:**
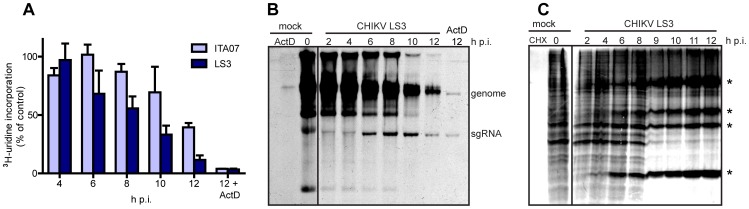
Transcriptional and translational shut-off induced by CHIKV. 293/ACE2 cells were infected with CHIKV LS3 or ITA07-RA1 at an MOI of 5, and at the indicated time points post infection metabolic labeling with ^3^H-uridine (A, B) or ^35^S-Cys and ^35^S-Met (C) was performed to analyze total RNA and protein synthesis, respectively. (A) Incorporation of ^3^H-uridine into viral and cellular RNA as measured by liquid scintillation counting of total RNA samples taken at various time points post infection. (B) ^3^H-uridine incorporation into viral and cellular RNA during CHIKV LS3 infection as detected by denaturing gel electrophoresis and fluorography. In control samples (ActD) 5 ug/ml Actinomycin D was added 30 min. prior to metabolic labeling to inhibit cellular transcription. (C) Synthesis of ^35^S-labeled viral and cellular proteins during CHIKV LS3 infection. The control lane labeled CHX contains proteins from cells treated with the translation inhibitor cycloheximide prior to metabolic labeling. CHIKV-specific proteins are indicated with a *.

To study CHIKV-induced transcriptional host shut-off, the incorporation of ^3^H-uridine into cellular and viral RNA was analyzed by metabolic labeling of infected 293/ACE2 cells at various time points post infection (MOI of 5). A strong reduction in the incorporation of ^3^H-uridine into RNA was observed at 10–12 h p.i. in cells infected with CHIKV LS3 or ITA07, as determined by liquid scintillation counting of total RNA samples (Fig. 3A). Inhibition of cellular transcription with actinomycin D for 30 min. prior to metabolic labeling at 12 h p.i. revealed the contribution of viral RNA synthesis to the total signal. Fluorographic detection of ^3^H-labeled RNA analyzed in denaturing gels also showed a decrease in cellular transcription during the course of the infection, while the synthesis of CHIKV RNA became clearly detectable by 6 h p.i (Fig. 3B). Transcriptional shut-off occurred around 10–12 h p.i. and was induced by CHIKV LS3 and ITA07 with similar kinetics, although LS3 seemed to act slightly faster. To examine CHIKV-induced translational shut-off, the synthesis of ^35^S-labeled viral and cellular proteins during the course of CHIKV LS3 infection was analyzed by metabolic labeling of infected 293/ACE2 cells with ^35^S-Met and ^35^S-Cys (Fig. 3C). A clear shut-off of host translation was observed 8–9 h p.i. Beyond 9 h p.i. the bulk of newly produced protein appears to be of viral origin, likely C, E1, E2 and their precursors (indicated with * in Fig. 3C). CHIKV ITA07 and LS3 induced translational host shut-off in a similar manner (only results obtained with LS3 are shown in Fig. 3C).

Both CHIKV ITA07-RA1 and the synthetic viruses established non-cytopathic persistent infections in C6/36 mosquito cells. All characterization experiments have been performed in both 293/ACE2 and Vero E6 cells, with similar results. For simplicity only the results for 293/ACE2 cells are shown, except for immunofluorescence experiments, which were done with Vero E6 cells as they had a more suitable morphology.

### Kinetics of RNA Synthesis of CHIKV ITA07-RA1 and the Synthetic Viruses

**Figure 4:**
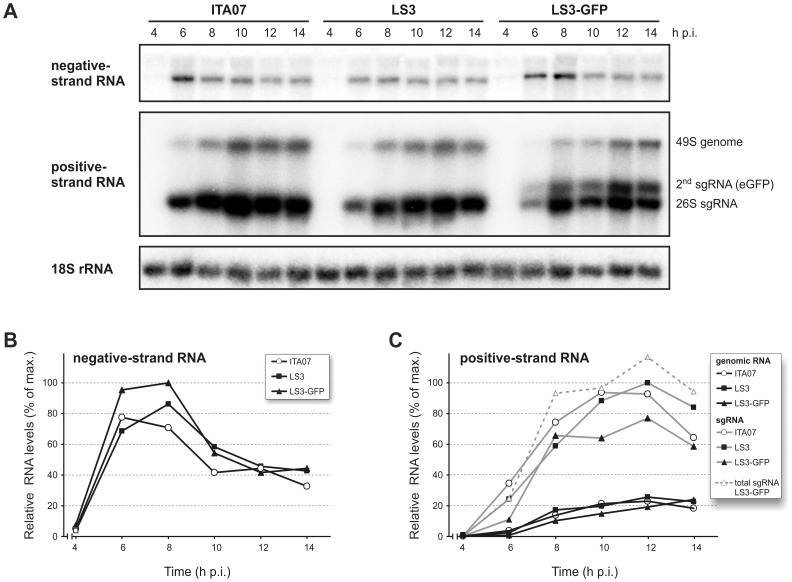
Accumulation of negative- and positive-strand CHIKV RNA in infected cells. (A) 293/ACE2 cells were infected with CHIKV LS3, LS3-GFP or ITA07-RA1 at an MOI of 5, total RNA was isolated at different time points post infection and strand-specific detection was performed with radioactively labeled oligonucleotides complementary to the 3′ end of either negative- (top panel) or positive-strand (middle panel) CHIKV RNA. Cellular 18S ribosomal RNA was probed as a loading control (lower panel). The positions of genomic RNA, the 26S sgRNA and the second eGFP-encoding sgRNA are indicated to the right of the middle panel. (B) Plot representing the kinetics of CHIKV negative-strand RNA accumulation, based on quantification of data from panel A. After correction for variations in sample loading based on the 18S rRNA signal, the relative abundance of the RNAs was determined by normalizing to the highest value observed (CHIKV LS3-GFP, 8 h p.i.). (C) Kinetics of CHIKV positive-strand RNA accumulation. The relative abundance of RNA was calculated as before, except that data were normalized to the value measured for LS3 sgRNA at 12 h p.i (100%). Genomic RNA levels are indicated with black lines, sgRNA levels with gray lines. The total level of both sgRNAs expressed by LS3-GFP is indicated with the gray dotted line.

The replication cycle of the synthetic viruses and ITA07-RA1 was characterized in more detail to assess whether the synthetic viruses behaved like their natural counterpart. The kinetics of RNA synthesis was analyzed by isolating total RNA from 293/ACE2 cells infected with CHIKV LS3, LS3-GFP, or ITA07-RA1 at various time points post infection. Negative- and positive-stranded RNAs were detected by hybridization with ^32^P-labeled oligonucleotide probes (Fig. 4A). Both negative- and positive-strand RNAs were readily detected in cells infected with the various strains starting at 6 h p.i. The negative-strand RNA was less abundant than the positive strand, it was easily detected relatively early in infection (Fig. 4A top panel, Fig. 4B), and appeared to decrease at later time points as has also been observed for other alphaviruses. This apparent decrease is probably not only due to degradation of minus strands, but at least partly due to a hampered detection caused by the large excess of positive-strand RNA present at late time points. This excess of positive-strand RNA competes with the radioactively labeled minus-strand specific probe. In support of this, we observed that mixing RNA isolated from CHIKV-infected cells at 6 h p.i. with *in vitro* transcribed positive-strand RNA reduced the amount of negative strand that could be detected (data not shown). Furthermore, when samples taken at 6 and 14 h p.i. were treated with single-strand-specific RNase A/T1 before hybridization, the negative-strand levels at the late time point were approximately 70% of that at 6 h p.i, instead of the approximately 50% in untreated samples (data not shown). Using a positive-strand-specific probe, both the 49S genomic and 26S sgRNA could be detected, and both RNAs accumulated until 12 h p.i (Fig. 4A middle panel, Fig. 4C). The ratio of genomic to sgRNA varied between 1∶3.5 and 1∶5.5 during the course of infection, similar to the ratios reported for Semliki forest virus and SINV [35]. The kinetics of RNA synthesis and RNA accumulation levels were similar in CHIKV LS3- and ITA07-RA1-infected cells. In cells infected with CHIKV LS3-GFP, the additional subgenomic RNA encoding the eGFP reporter gave rise to an extra band above the 26S RNA band, and its expression level was calculated to be approximately half of that of the 26S RNA. The individual levels of the two sgRNAs expressed by CHIKV LS3-GFP were lower than those of ITA07 or LS3, but their combined abundance was comparable to that of the viruses expressing a single sgRNA (Fig. 4C).

### CHIKV Protein Synthesis and dsRNA Accumulation in Cells Infected with ITA07-RA1 or the Synthetic Viruses

**Figure 5:**
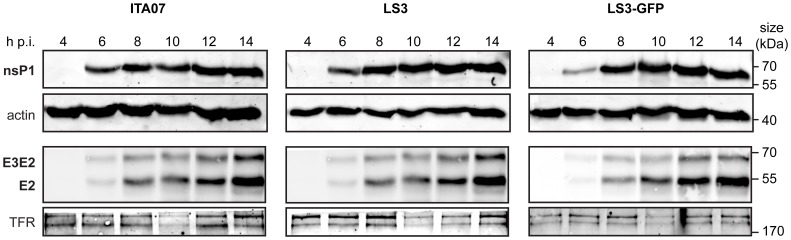
Western blot analysis of CHIKV nsP1 and E2 expression at different time points post infection. 293/ACE2 cells were infected with CHIKV ITA07, LS3 or LS3-GFP at an MOI of 5. At the indicated time points cells were lysed, proteins were separated by SDS-PAGE and viral proteins were detected by Western blotting. The anti-E2 antiserum also recognized the E3E2 (p62) precursor of E2. Actin and the transferrin receptor were used as loading controls.

To monitor viral protein expression, 293/ACE2 cells were infected with CHIKV LS3, LS3-GFP, or ITA07-RA1 and total protein was isolated at various time points post infection. These samples were analyzed by Western blotting with antisera against the nonstructural proteins nsP1 and nsP4, and the structural protein E2. Expression of nsP1, E2, and the E3E2 precursor could be detected as early as 6 h p.i. and the proteins accumulated over time, reaching a plateau around 12 h p.i. (Fig. 5). The RdRp nsP4 could not be detected in infected cells using a CHIKV nsP4-specific antiserum capable of detecting the purified bacterially expressed protein. This was probably due to the low affinity of the antibody, the low expression level and relative instability of nsP4 in infected cells, as was also observed for other alphaviruses [36]. In addition, a quantitative proteomics study on CHIKV-infected cells also suggested that at 10 h p.i. the amount of nsP4 was at least 200-fold lower than that of nsP1 (Treffers, Tas, de Ru, van Veelen, Snijder and van Hemert, manuscript in preparation).

**Figure 6:**
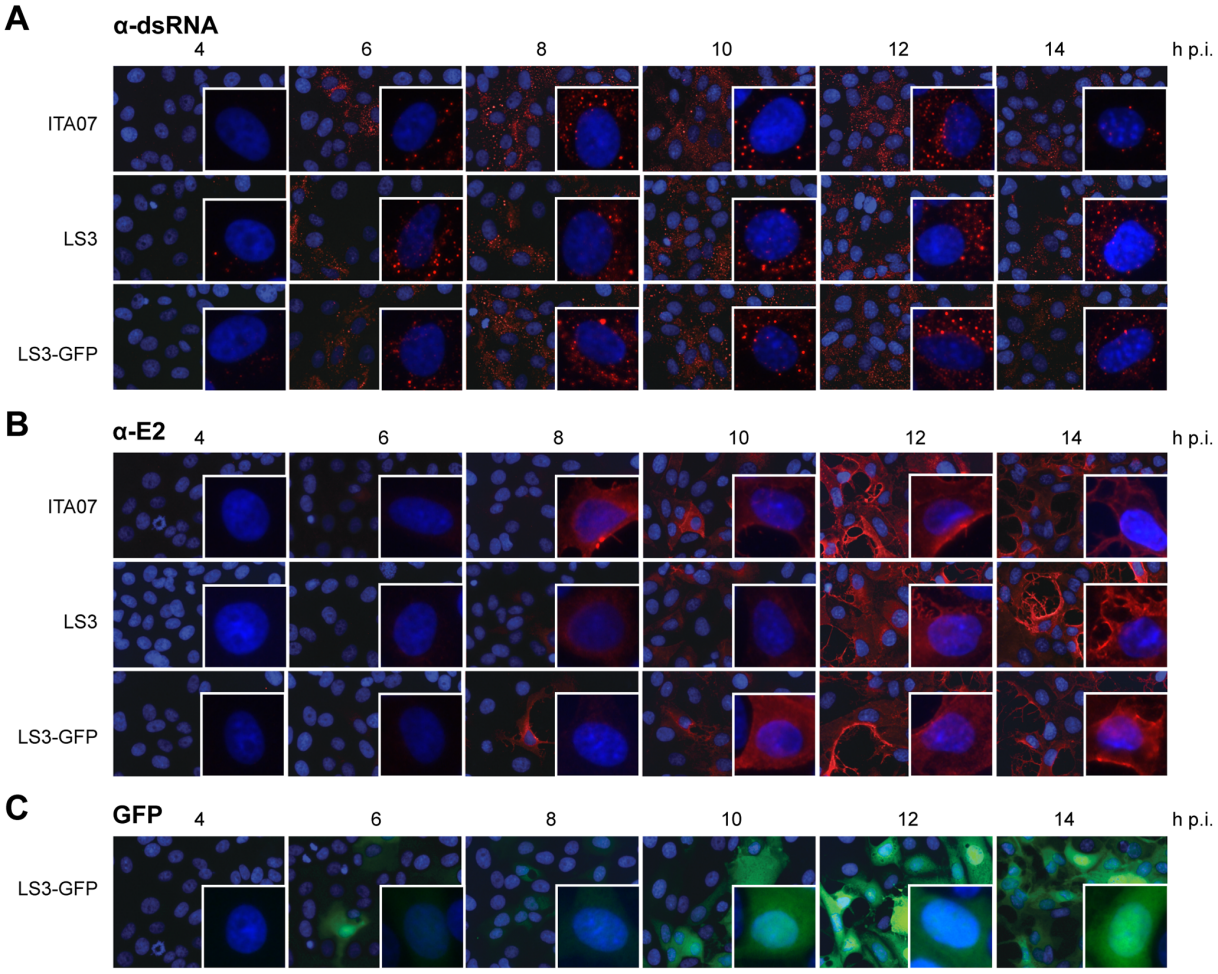
Immunofluorescence analysis of dsRNA and E2 expression in time. Vero E6 cells grown on coverslips were infected with CHIKV ITA07, LS3 or LS3-GFP at an MOI of 5. At the indicated time points the coverslips were fixed and stained with antibodies specific for dsRNA (A) or E2 (B). (C) eGFP fluorescence in CHIKV LS3-GFP infected cells (green). Nuclei (blue) were visualized by Hoechst staining.

Indirect immunofluorescence analysis of Vero E6 cells infected with CHIKV LS3, LS3-GFP, or ITA07-RA1 at various time points showed that the localization and expression kinetics of E2 and dsRNA were similar for the natural isolate and the synthetic viruses (Fig. 6). Double-stranded RNA, which is assumed to be generated during replication of CHIKV in infected cells [37], could be detected as early as 4 h p.i. and remained clearly visible throughout the infection. The dsRNA localized to foci throughout the cytoplasm. The E2 protein could be detected from 6 h p.i. onwards with maximum expression reached by 12 h p.i. The E2 protein mainly localized to the plasma membrane of infected cells. eGFP produced by the reporter virus was visible from 6 h p.i. onwards, reaching a maximum level around 12 h p.i. (Fig. 6C).

### Neutralization of CHIKV LS3 by a Monoclonal Antibody Raised Against ITA07-RA1

**Figure 7:**
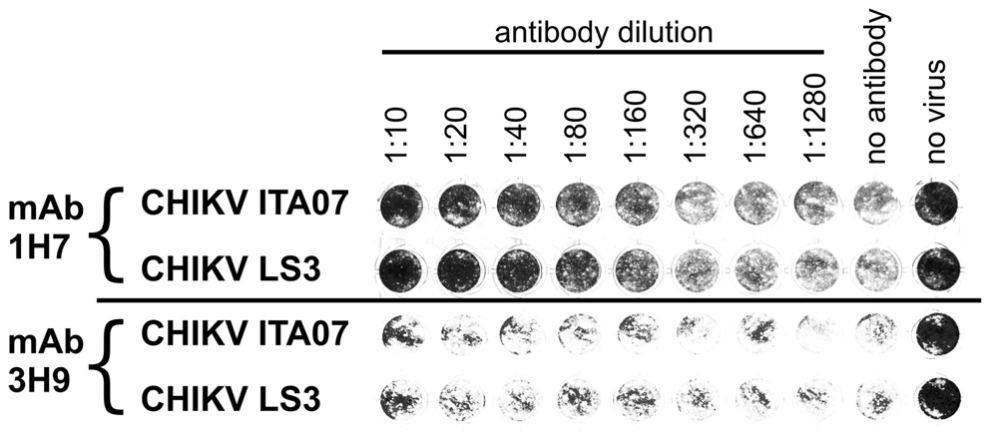
Neutralization of CHIKV ITA07 and LS3 by mouse monoclonal antibodies raised against ITA07-RA1. 100 PFU of the CHIKV strains were incubated with serially diluted antibodies in 96-well clusters containing confluent monolayers of VeroE6 cells. After 2 days the wells were fixed with formaldehyde and stained with crystal violet. Either the neutralizing antibody 1H7 or non-neutralizing control antibody 3H9 were used.

CHIKV LS3 and ITA07-RA1 were compared in a neutralization assay using the neutralizing monoclonal antibody 1H7 that was raised in mice against CHIKV ITA07-RA1 virions, and appears to recognize a linear epitope in E2 [31]. The non-neutralizing mAb 3H9 was used as a control. Both the natural isolate and CHIKV LS3 were neutralized with similar characteristics by 1H7, while their infectivity was not affected by 3H9 (Fig. 7).

### The Synthetic Viruses Cause Lethal Infections in a Mouse Model

**Figure 8:**
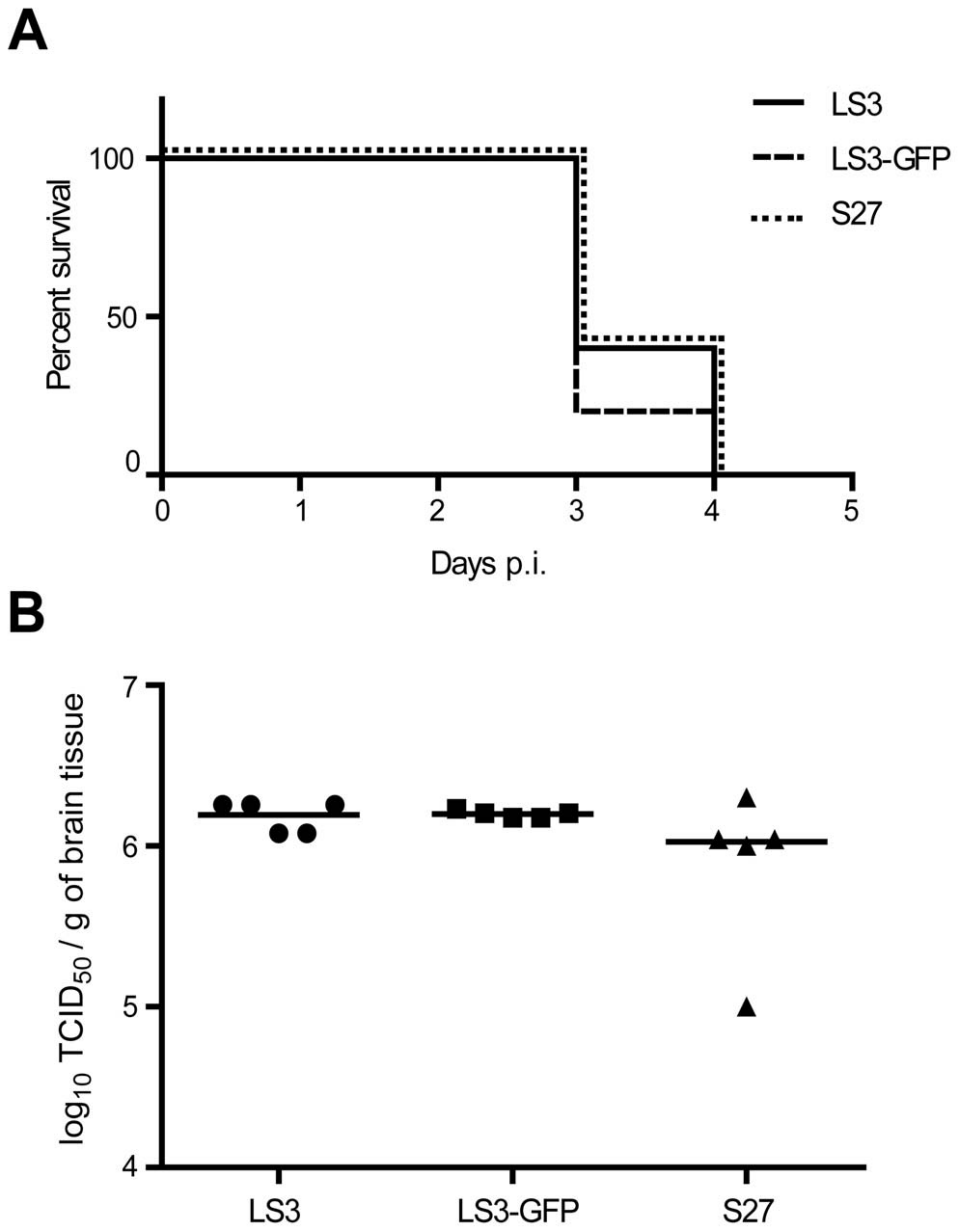
Replication of synthetic CHIKV strains *in vivo*. (A) Survival of mice after intraperitoneal injection with 100 TCID_50_ of CHIKV LS3, LS3-GFP or S27 (5 mice per group). (B) CHIKV titers (in TCID_50_ equivalents per mg of tissue) in the brains of the mice infected with the 3 CHIKV strains.

Newborn mice are highly susceptible to CHIKV infection and they develop symptoms as lethargy, dragging of hind limbs, flaccid paralysis, and reduced weight gain [38]. 12-day old mice were injected intraperitoneally with 100 TCID_50_ of CHIKV LS3, LS3-GFP or prototype strain S27 as a control. The animals were euthanized when their humane end points were reached 3 or 4 days post infection and viral RNA levels in brain tissue were analyzed (Fig. 8). Both synthetic viruses behaved like the natural isolate *in vivo,* causing lethal infections with similar kinetics (Fig. 8A). In addition, the viral titers in the brains of CHIKV S27-infected mice were similar to those of mice infected with the synthetic viruses (Fig. 8B).

### Sensitivity to Antiviral Compounds

**Figure 9:**
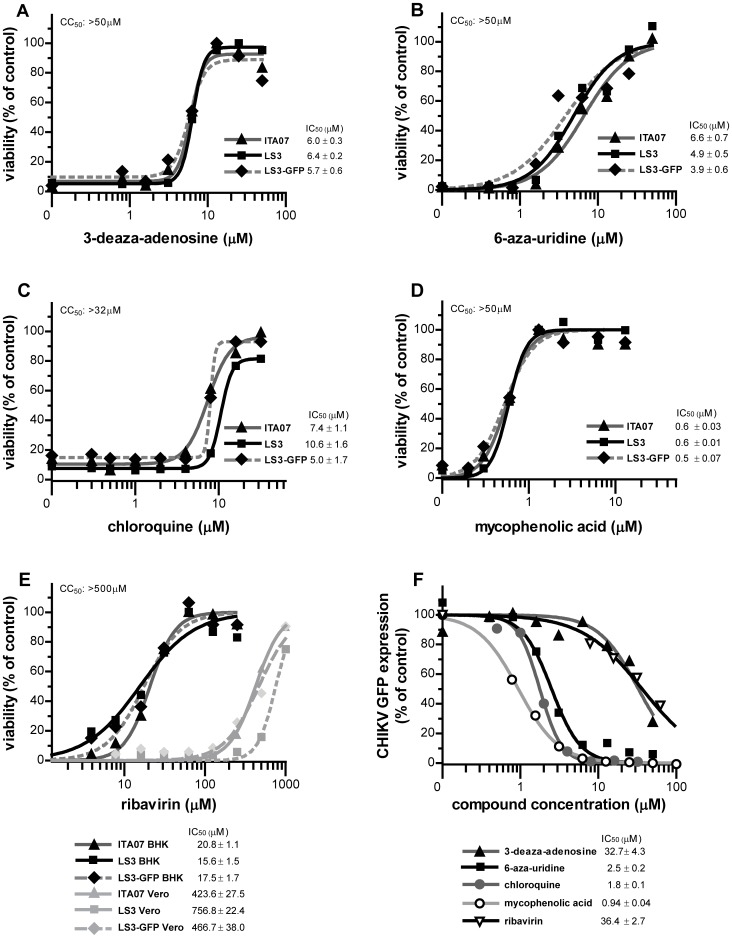
Effect of antiviral compounds on the replication of various CHIKV strains. Dose dependent reduction of CHIKV-induced CPE by (A) 3-deaza-adenosine, (B) 6-aza-uridine, (C) chloroquine and (D) mycophenolic acid in Vero E6 cells infected with CHIKV strains ITA07-RA1, LS3 and LS3-GFP (MOI 0.005). (E) Antiviral effect of ribavirin on CHIKV replication in BHK-21 (black lines) and Vero E6 cells (gray lines). Cell viability was normalized to untreated uninfected cells (100%). The 50% cytotoxic concentration (CC_50_) of the compounds is indicated in the top left of each panel. (F) Dose-response curves showing the effect of five antiviral compounds on the eGFP expression in Vero E6 cells infected with CHIKV LS3-GFP (MOI 0.05) at 42 h p.i. Values were normalized to eGFP fluorescence in untreated infected cells (100%).

To evaluate their suitability for analyzing the potency and mechanism of action of antiviral compounds, the sensitivity of CHIKV LS3 and LS3-GFP to a number of such compounds was determined and compared to ITA07-RA1. Cyclosporin A, which through its effect on cellular cyclophilins inhibits the replication of a variety of viruses, had no specific effect on CHIKV replication, not even at a (cytotoxic) dose of 32 µM (data not shown). The compounds 3-deaza-adenosine, 6-aza-uridine, chloroquine, and mycophenolic acid were tested in CPE reduction assays with Vero E6 cells infected at an MOI of 0.005 and analyzed 4 days p.i. They were all found to inhibit CHIKV replication with IC_50_s in the low micromolar range and with minimal cytotoxicity (Fig. 9A–D). No substantial differences were observed between the IC_50_ values calculated for ITA07-RA1, LS3 and LS3-GFP. The four compounds also clearly reduced eGFP reporter gene expression in Vero E6 cells infected with CHIKV LS3-GFP (Fig. 9F). Slightly lower IC_50_ values were obtained for 6-aza-uridine and chloroquine, and a significantly higher IC_50_ was observed for 3-deaza-adenosine in this assay, compared to the CPE-based assay. This might be due to the mode of action of 3-deaza-adenosine and/or due to differences in experimental set-up compared to the CPE-based assay (MOI 0.05 vs. 0.005; measurement 42 h p.i. vs. 4 d p.i). Ribavirin is a known inhibitor of CHIKV replication, but in our CPE reduction assay with Vero E6 cells it was not very effective in inhibiting replication of the various strains, as IC_50_ values of over 400 µM were obtained (Fig. 9E, gray lines). This is likely due to the inefficient conversion of ribavirin to its active phosphorylated form in Vero E6 cells [39]. Therefore, we have also analyzed the effect of ribavirin in a 2-day CPE reduction assays with BHK-21 cells, which are able to metabolize ribavirin [40], [41] and found IC_50_s of 15–21 µM for the various strains. Clinical isolate NL10/152 was also included in the assays and appeared to be somewhat more sensitive to the antiviral compounds than LS3 and ITA07-RA1. However, the slower replication kinetics of this strain made it impossible to directly compare NL10/152 and LS3 in the same CPE reduction assays, despite the fact that virus yields and cytopathic effect of NL10/152 and LS3 were comparable (data not shown).

## Discussion

The massive CHIKV outbreaks that have been occurring in Asia and the Indian Ocean region since 2005 are associated with the emergence of strains with the A226V substitution in the E1 glycoprotein, which allowed their transmission by a novel mosquito vector, *Aedes albopictus* [8]–[13]. These East-Central-South African lineage-derived strains even appear to be replacing the Asian lineage CHIKV strains that have been endemic in the region for decades. Since the 1980s, the geographic distribution of *Aedes albopictus* has dramatically expanded and now also includes large parts of the USA and several European countries. This creates concern for locally transmitted outbreaks in Europe and the USA, which could be initiated by viraemic travelers arriving from countries where CHIKV is endemic, like India and Indonesia. Locally transmitted CHIKV infections have indeed already been reported from Italy in 2007 and France in 2010 [15], [16] and recent studies suggest that also the USA is at risk for locally transmitted CHIKV outbreaks [42], [43]. Besides its large medical and societal impact in endemic countries, the increased risk of CHIKV outbreaks in Europe and the USA underlines the importance of studying the replication of this important human pathogen and its interactions with the host to develop safe and effective vaccines and antiviral therapy.

Infectious cDNA clones have proven to be important tools to study many aspects of the viral life cycle, and molecular clones of a variety of natural isolates have been instrumental in several recent CHIKV studies [17]–[23]. The existing CHIKV molecular clones can be considered to be derived from a single genome (or fragments of single genomes) out of the whole spectrum of viruses present in a CHIKV quasispecies population. In contrast, most of the complete CHIKV genome sequences that have been deposited in GenBank represent the consensus (or master sequence) of a viral quasispecies population. The diversity (and evolution) of a CHIKV quasispecies population has probably been shaped by the characteristics of the individual host and the specific tissue source (serum) from which it was isolated. For Ross River virus it was observed that the level of intrahost genetic variation in patient serum samples, was considerably larger than that observed at the epidemiological scale, which can be explained by the purifying selection for replication in both arthropod and vertebrate hosts [24]. Advances in sequencing techniques now allow a more detailed view on quasispecies diversity and intrahost evolution, and also for CHIKV a recent study has provided more insight into quasispecies dynamics and the effect of purifying selection by host alternation [44]. A link was observed between increased fitness as a result of alternating passaging and reduced quasispecies complexity, which restricted adaptability to novel selective pressures like antiviral treatment or antibody-mediated neutralization [44].

Individual CHIKV isolates or molecular clone derived viruses could have their specific properties in terms of replication kinetics, vector specificity, dissemination within the host, virulence, virus-host interactions or sensitivity to antiviral compounds. We were interested in studying the general characteristics of the life cycle and virus-host interactions of the E1-226V CHIKV strains that were circulating during the 2005–2009 outbreaks. Therefore, we have constructed a fully synthetic cDNA clone, CHIKV LS3, based on the consensus sequence of the aligned genomes of these recent E1-226V isolates, rather than on a single genome from a clinical isolate. In addition, a variant that expresses the eGFP reporter gene under control of a (duplicated) subgenomic promoter was created (CHIKV LS3-GFP). The current possibilities of gene synthesis allowed the design of these clones *in silico*, with sequences tailored to our requirements, e.g. already containing a reporter gene under control of a duplicated subgenomic promoter and including (translationally silent) mutations to create restriction sites that facilitate cloning and reverse genetics studies.

Alignment of all 148 complete CHIKV genomes that were in GenBank by June 2013 yielded a consensus sequence that differed only at 3 nucleotide positions from the sequence of LS3 that was designed 3 years earlier. These were position 7,435 at which we introduced a translationally silent restriction site (G7435A), a synonymous U→C substitution at position 3,397, and position 10,670, which is a C in 68% of all deposited genomes (strains with E1-226A), while the remaining (E1-226V) strains have a U at this position. An interesting observation was that 6% of the sequenced CHIKV strains, including the prototype strains S27 and Ross, contain an arginine codon instead of the opal stop codon that is present between the nsP3- and nsP4-coding regions of most CHIKV isolates. The presence or absence of this opal codon might be influenced by the passage history of the isolate as has been observed for other alphaviruses [45]. This might also explain why the sequence of the original clinical isolate of LR2006-OPY1 (Genbank DQ443544.2) contains the opal termination codon near the end of the nsP3 coding region, while the infectious clone of this strain (Genbank EU224268.1) contains an arginine codon at this position.

To assess whether the synthetic viruses are representative models, their characteristics were compared to those of the natural strain ITA07-RA1. Like the natural isolate, the synthetic viruses caused cytopathic infections in Vero E6 and 293/ACE2 cells (Fig. 2E), whereas non-cytopathic persistent infections were observed in the mosquito cell line C6/36. In vertebrate cells all strains caused a shut-off of cellular translation around 8–9 h p.i. and a strong inhibition of cellular transcription by 10–12 h p.i. The accumulation of negative- and positive-strand viral RNA, the kinetics of non-structural and structural viral protein expression, as well as the growth kinetics and plaque morphology of the synthetic viruses were indistinguishable from those of CHIKV ITA07-RA1 (Fig. 2–6). In addition, the synthetic viruses caused lethal infections in 12-day old mice, with virus spreading to the brain, as observed for natural isolates (Fig. 8). Although this demonstrates that the synthetic viruses replicate *in vivo*, this mouse model does not allow comparison of strains for more subtle differences in virulence and pathogenesis. The genomic stability of CHIKV LS3-GFP was assessed and after 3 passages its (consensus) sequence was found to be identical to the original *in silico* designed sequence. The expression of the eGFP reporter gene was stable for at least 10 passages, making the synthetic viruses suitable tools for high-throughput screens for antiviral compounds, (reverse genetics) studies into their mechanism of action, and systematic functional genomics screens for host factors affecting CHIKV replication.

To evaluate whether CHIKV LS3 and LS3-GFP are suitable to analyze the potency and mechanism of action of antiviral compounds, their sensitivity to a number of such compounds was determined and compared to ITA07-RA1. The lysosomotropic agent chloroquine and nucleoside analog 6-aza-uridine inhibited the replication of the synthetic viruses and natural isolates with IC_50_s that were in the same range and comparable to values previously reported by others [46]–[51]. The inhibitory effect of chloroquine on the replication of many viruses including alphaviruses has been known for decades. For CHIKV it is a useful reference compound in cell-based studies, but a small scale clinical trial on the island of La Reunion suggested it is not effective in the treatment of CHIKV infections in patients [46]. The nucleoside analog 6-aza-uridine has previously been reported to inhibit the replication of a variety of viruses, including CHIKV [47], [51]. The compound could interfere with cellular UTP metabolism and may be incorporated into CHIKV RNA, leading to chain termination and/or increased error frequency, ultimately resulting in ‘error catastrophe’. Mycophenolic acid is a non-competitive inhibitor of inosine monophosphate dehydrogenase (IMPDH), causing a depletion of the intracellular guanosine pool. It is a known inhibitor of various viruses, including CHIKV [47], [52]. Ribavirin is a synthetic nucleoside analog with broad spectrum antiviral effect due to potential effects on the cellular IMPDH enzyme, viral RNA synthesis and capping [39]. However, not all cell lines are able to perform the necessary conversion of this compound to its active phosphorylated form, explaining the contradictory reports on the antiviral activity of this compound [40], [41], [53]. In our hands, ribavirin inhibited CHIKV replication in BHK-21 cells with an IC_50_ of around 18 µM, while it was hardly effective in Vero E6 cells, with IC_50_ values of over 400 M. The IC_50_ that we obtained with BHK-21 cells is in the same range as those previously reported for the antiviral effect of ribavirin on CHIKV replication [47], [51]. Cyclosporin A, which through its effect on the cellular cyclophilins, inhibits the replication of a variety of viruses (for recent review see [54]), had no effect on CHIKV replication. We identified 3-deaza-adenosine as a novel inhibitor of CHIKV replication with an IC_50_ of approximately 6 µM and a CC_50_>50 µM. This compound has previously been identified as inhibitor of a broad spectrum of viruses, although many other +RNA viruses appeared to be rather insensitive or not affected at all (reviewed in [55]). The antiviral activity of 3-deaza-adenosine was attributed to its inhibitory effect on the cellular enzyme S-adenosylhomocysteine hydrolase, leading to an accumulation of S-adenosylhomocysteine, which inhibits S-adenosylmethionine-dependent methylation reactions [55]. In this manner the enzyme plays a key role in S-adenosylmethionine-dependent methylation reactions and inhibition of viral methylation reactions (e.g. of viral RNA) apparently can be achieved at compound concentrations that do not notably interfere with cellular methylation reactions. Our observation warrants a more detailed analysis of the mode of action of 3-deaza-adenosine and analogs, also to evaluate their potential for use in antiviral therapy to treat CHIKV infections. Overall, no large differences were observed between the IC_50_ values calculated for ITA07-RA1, LS3 and LS3-GFP, indicating that the synthetic viruses are suitable for use in antiviral screens. For most compounds, a faster and simpler assay with CHIKV LS3-GFP reporter virus showed a good dose-dependent response that correlated well with results obtained in the CPE-based assay.

Clinical isolate NL10/152 exhibited slightly slower replication kinetics and appeared to be more sensitive to antiviral compounds than ITA07 and the synthetic viruses. Differences in the sensitivity to antiviral compounds among clinical isolates is not an uncommon phenomenon. NL10/152 differs at 7 amino acid positions from LS3 and it will be interesting to determine the contribution of these mutations, in particular the R88S substitution in nsP4, to the slower replication kinetics (and higher sensitivity to antivirals).

Taken together the detailed characterization of the CHIKV replication cycle at the molecular level demonstrated that our new synthetic consensus-based viruses behave like natural isolates and are suitable tools to study various aspects of the CHIKV life cycle, which should ultimately provide a basis for the development of antiviral therapy.

## Supporting Information

Table S1 **Comparison of CHIKV LS3 with the genome sequences of various closely related natural isolates.** Only differences between LS3 and each of the other strains are summarized. Dots indicate that the nucleotide at that position is identical to that at the corresponding position in the sequence of LS3. Genomes were aligned with MAFFT and analyzed in Jalview. Numbering is based on the sequence of LR2006_OPY1 (and is equal to LS3 numbering). The nucleotide at position 10670 (indicated in gray) determines whether the strain has the A226V mutation in the E1 protein. Strains with a T at this position have the A226V mutation. Differences not included in the comparison are the 35 nt, 5 nt and 23 nucleotides that are missing from the 3’UTR of the sequences of DRDE-07, D570/06 and ITA07-RA1, respectively. The missing first 19 nt, missing last 13 nt and the insertion of an A after position 11564 in the sequence of IND-06-AP3 were also not included in this comparison.
(PDF)
