# Supplementary material for: Characterization of Synthetic Chikungunya Viruses Based on the Consensus Sequence of Recent E1-226V Isolates
Source: PLoS One. 2013 Aug 1;8(8):e71047. doi: 10.1371/journal.pone.0071047 (PMC3731263; doi:10.1371/journal.pone.0071047)
Supplement: Table S1 — Comparison of CHIKV LS3 with the genome sequences of various closely related natural isolates. Only differences between LS3 and each of the other strains are summarized. Dots indicate that the nucleotide at that position is identical to that at the corresponding position in the sequence of LS3. Genomes were aligned with MAFFT and analyzed in Jalview. Numbering is based on the sequence of LR2006_OPY1 (and is equal to LS3 numbering). The nucleotide at position 10670 (indicated in gray) determines whether the strain has the A226V mutation in the E1 protein. Strains with a T at this position have the A226V mutation. Differences not included in the comparison are the 35 nt, 5 nt and 23 nucleotides that are missing from the 3’UTR of the sequences of DRDE-07, D570/06 and ITA07-RA1, respectively. The missing first 19 nt, missing last 13 nt and the insertion of an A after position 11564 in the sequence of IND-06-AP3 were also not included in this comparison. (PDF) [file pone.0071047.s001.pdf]

Table S1. Comparison of CHIKV LS3 with the genome sequences of various closely related natural isolates. *Only differences between LS3 and each of the other strains are summarized. Dots indicate that the nucleotide at that position is identical to that at the corresponding position in the sequence of LS3. Genomes were aligned with MAFFT and analyzed in Jalview. Numbering is based on the sequence of LR2006_OPY1 (and is equal to LS3 numbering). The nucleotide at position 10670 (indicated in gray) determines whether the strain has the A226V mutation in the E1 protein. Strains with a T at this position have the A226V mutation. Differences not included in the comparison are the 35 nt, 5 nt and 23 nucleotides that are missing from the 3'UTR of the sequences of DRDE-07, D570/06 and ITA07-RA1, respectively. The missing first 19 nt, missing last 13 nt and the insertion of an A after position 11564 in the sequence of IND-06-AP3 were also not included in this comparison.*

	nt position / strain	LS3	IND-06-AP3	CHIK31	IND-MH51	LR2006_OPY1	DRDE-07	D570/06	ITA07-RA1
5'-UTR	1	**A**	-	.	T<br>T	.	.	.	T
nsP1	358	**T**	.	.	.	C	.	C	.
	459	**A**	.	.	.	C	.	C	.
	499	**T**	.	.	C	.	.	.	.
	568	**C**	.	.	.	.	.	T	.
	627	**T**	.	.	.	.	C	.	.
	757	**T**	.	.	.	.	.	.	C
	764	**G**	.	.	.	.	.	.	A
	862	**G**	C	.	.	.	.	.	.
	985	**T**	.	.	.	.	.	.	C
	1016	**A**	.	.	.	.	T	.	.
	1052	**G**	.	.	.	R	.	.	.
	1203	**T**	.	.	.	C	.	C	.
	1247	**T**	.	.	C	.	.	.	.
	1381	**T**	.	.	.	.	.	.	G
	1603	**C**	.	.	.	.	T	.	.
nsP2	2014	**A**	.	.	.	.	.	.	G
	2944	**G**	.	.	.	.	A	.	.
	3325	**C**	.	.	.	.	.	.	T
	3397	**T**	.	.	.	.	.	.	C
	3481	**C**	.	.	.	.	.	.	T
	3724	**T**	.	.	.	A	.	A	.
nsP3	4167	**A**	.	.	.	R	.	.	.
	4300	**T**	.	.	C	.	.	.	.
	5049	**G**	.	.	.	K	.	.	.
	5122	**T**	.	.	.	.	.	C	.
	5202	**C**	.	.	.	.	.	.	T
	5248	**C**	.	.	.	T	.	T	.
	5377	**G**	.	.	.	.	.	.	A
	5490	**G**	.	A	.	.	.	.	.
	5491	**C**	.	T	.	.	.	.	.
nsP4	5744	**C**	.	.	.	.	.	.	T
	6397	**C**	.	.	.	.	T	.	.
	6547	**G**	.	.	.	A	.	A	.
	6706	**C**	.	T	.	.	.	.	.
	7435	**G**	A	A	A	A	A	A	A
	7450	**C**	.	.	.	.	.	.	T
C	7633	**T**	.	.	.	C	.	C	.
	7645	**G**	.	A	.	.	.	.	A
	7983	**C**	.	.	.	.	.	.	T
	8127	**C**	.	.	.	.	.	.	T
E3	8385	**C**	.	.	.	.	T	.	.
E2	8910	**T**	.	.	.	C	.	C	.
	8985	**T**	.	.	.	.	A	.	.
	9114	**A**	.	.	.	.	G	.	.
	9207	**T**	.	.	A	.	.	.	.
	9633	**T**	.	.	.	C	.	C	.
	9681	**G**	.	.	.	.	.	.	A
E1	10004	**T**	.	.	.	.	C	.	.
	10314	**T**	.	.	C	C	.	C	.
	10377	**T**	.	.	.	.	.	.	C
	10670	**T**	C	C	C	.	.	.	.
	10743	**A**	.	.	.	G	.	G	.
	11127	**T**	.	.	.	C	.	C	.
	11256	**T**	.	.	.	.	.	C	.
3'-UTR	11360	**T**	.	.	.	.	C	.	.
	11499	**G**	T	.	.	.	.	.	.
	11600	**C**	.	.	.	.	.	.	T
	11640	**A**	.	.	.	.	.	.	G
	11723	**T**	.	-	.	.	.	.	.
	11762	**T**	.	.	.	.	-	.	.
	11763	**C**	.	.	.	.	-	.	.
	11765	**C**	.	.	.	.	T	.	.
	11770	**C**	.	.	.	.	A	.	.
	11776	**G**	.	.	.	.	C	.	.
	11784	**G**	-	.	.	.	-	.	.
**total nt difference**			5	7	9	16	16	16	21
